# Identification of novel transaminases from a 12-aminododecanoic acid-metabolizing *Pseudomonas* strain

**DOI:** 10.1111/1751-7915.12278

**Published:** 2015-04-24

**Authors:** Matthew Wilding, Ellen F A Walsh, Susan J Dorrian, Colin Scott

**Affiliations:** 1 CSIRO Land and Water Flagship, Black MountainCanberra, ACT, 2601, Australia; 2 CSIRO Food and Nutrition Flagship, Black MountainCanberra, ACT, 2601, Australia; 3 Research School of Chemistry, Australian National UniversityCanberra, ACT, 2601, Australia

## Abstract

A *Pseudomonas* species [*Pseudomonas* sp. strain amino alkanoate catabolism (AAC)] was identified that has the capacity to use 12-aminododecanoic acid, the constituent building block of homo-nylon-12, as a sole nitrogen source. Growth of *Pseudomonas* sp. strain AAC could also be supported using a range of additional ω-amino alkanoates. This metabolic function was shown to be most probably dependent upon one or more transaminases (TAs). Fourteen genes encoding putative TAs were identified from the genome of *Pseudomonas* sp. AAC. Each of the 14 genes was cloned, 11 of which were successfully expressed in *Escherichia coli* and tested for activity against 12-aminododecanoic acid. In addition, physiological functions were proposed for 9 of the 14 TAs. Of the 14 proteins, activity was demonstrated in 9, and of note, 3 TAs were shown to be able to catalyse the transfer of the ω-amine from 12-aminododecanoic acid to pyruvate. Based on this study, three enzymes have been identified that are promising biocatalysts for the production of nylon and related polymers.

## Introduction

There has been a substantial increase in the impact that biocatalysis has had on the chemical manufacturing industry, to the extent that for some reaction chemistries, biocatalysis is the preferred option. Many of the commercial successes of biocatalytic processes have been in the manufacture of fine chemicals and pharmaceuticals, reboxetine, pregabalin and sitagliptin, for example (Martinez *et al*., 2008; Savile *et al*., 2010; Hayes *et al*., 2011; Bornscheuer *et al*., 2012). However, there has also been considerable technical success in developing processes for the production of bulk chemicals, especially for polymers such as polyesters or functionalized polyethylene glycols (Gross *et al*., 2010; Bhatia *et al*., 2011).

**Fig 1:**
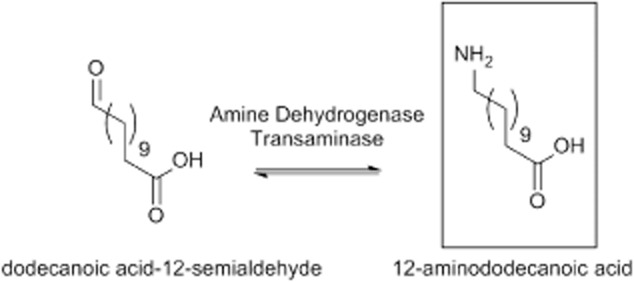
Potential biocatalysts for the production of 12-aminododecanoic acid.

Global nylon-12 shortages in recent years (Advisen, 2013) have afforded new opportunities within the materials field. The hazards associated with the manufacture of these materials are documented (Thiemens and Trogler, 1991) and represent an opportunity for alternative biocatalytic methods (Lithner *et al*., 2011). However, the synthesis of these ω-amino acids represents something of a challenge for biocatalysts. While short-chain ω-amino acids, such as β-alanine and 4-aminobutyrate, have well-known biological roles (Matthews and Traut, 1987; Watanabe *et al*., 2002), long-chain ω-amino acids (e.g. 12-aminododecanoic acid) have no known physiological role and it is unlikely that enzymes have evolved to interact with such molecules. On the other hand, non-physiological promiscuous catalytic activities are relatively common (Copley, 2003; Khersonsky *et al*., 2006), and it is possible that an appropriate promiscuous function could be used in the biocatalytic synthesis of 12-aminododecanoic acid, or as a starting point for directed evolution (Turner, 2009). For example, either a transaminase (TA) or an amine dehydrogenase could possess the capacity to aminate dodecanoic acid-12-semialdehyde *via* a non-physiological enzymatic activity (Fig. 1).

There are a small number of reported methods to access these ω-semialdehyde intermediates, with dehydrogenase-catalyzed oxidations of ω-hydroxycarboxylic acids reported in several species (Dickinson and Dack, 2001; Lu *et al*., 2010). In addition, during the preparation of this manuscript, an elegant method to functionalize aliphatic fatty acids at the omega position was reported by Schrewe *et al*, (2013).

## Results and discussion

### *Pseudomonas* sp. strain AAC can use 12-aminododecanoic acid as a nitrogen source

A growth-linked screen was conducted to identify a bacterium that possessed an enzyme with suitable properties for 12-aminododecanoic acid production, whereby organisms were grown on nitrogen-deficient M9 medium that had been supplemented with 12-aminododecanoic acid. Forty-seven bacterial strains, taken from the CSIRO Land and Water Flagship strain collection (Clinton *et al*., 2011) (formerly known as the CSIRO Entomology strain collection), were screened and a bacterium was identified that could grow on 12-aminododecanoic acid as a sole nitrogen source. The bacterium that grew was isolated and was identified as a *Pseudomonas* sp. by 16s rDNA sequencing (99% identity with *Pseudomonas nitroreductans* strain T2; accession no. JF441245.1). Growth assays using β-alanine, γ-aminobutyrate, δ-aminovaleric acid and ε-aminocaproic acid as sole nitrogen sources revealed that this bacterium could use a broad range of ω-aminoalkanoates, and so the bacterium was named *Pseudomonas* sp. strain AAC (for amino alkanoate catabolism).

Both TAs and amine dehydrogenases perform reversible reactions and either could, in principle, perform the amination of dodecanoic acid semialdehyde (Fig. 1). To determine the enzymatic mechanism used by *Pseudomonas* sp. strain AAC to access the nitrogen from 12-aminododecanoic acid, cell lysate was tested for activity. Transformation of 12-aminododecanoic acid was found to proceed when the cell-free extract was supplemented with a mixture of α-ketoglutarate and pyruvate (i.e. common TA co-substrates), but not in their absence (data not shown). This observation was consistent with a TA catalyzed reaction.

### *Pseudomonas* sp. strain AAC genome possesses 14 TAs

The genome of *Pseudomonas* sp. AAC was sequenced (Accession number: JNCW00000000; Beijing Genome Institute, BGI, Beijing). The total genome sequence was 7.07 Mbp in length, had a GC content of 67% and 6097 genes.

The protein2genome model alignment in Exonerate (Slater and Birney, 2005) was used to identify the genes encoding potential TAs in the *Pseudomonas* sp. strain AAC genome. *Escherichia coli* GabT (4-aminobutyrate TA) was selected as the query sequence for analysis. Although there were no reports in the literature that the protein could catalyse the desired reaction, it had been shown to act on ω-aminoalkanoates of up to C7 in length (Yonaha *et al*., 1985). Following analysis of the genomic DNA, 14 potential homologues were identified, ranging in sequence identity (versus GabT) from 4% to 74% (Table 1).

**Table 1:** TAs identified in *Pseudomonas* sp. strain AAC

Protein,^a^ proposed function^b^	Protein size (kDa)^c^	% Identity to GabT	Expression conditions	Activity
KES22976, 4-aminobutyrate aminotransferase	44.6	74	Rosetta 2-DE3, 15°C	Cadaverine
KES23551, 4-aminobutyrate aminotransferase	45.2	73	BL21-DE3, 15°C	4-Aminobutyrate
KES24870, acetylornithine aminotransferase	43.5	31	BL21-DE3, 37°C	N-Acetyl-L-ornithine
KES25161, hypothetical protein	47.5	30	BL21-DE3, 37°C	β-amino acid
KES22989, taurine–pyruvate aminotransferase	60.5	30	Failed	
KES21039, 2,4-diaminobutyrate 4-aminotransferase	50.4	30	Rosetta 2-DE3, 15°C	β-alanine/aminoisobutyrate
KES23360, hypothetical protein	49.3	27	BL21-DE3, 15°C	Cadaverine
KES21385, alanine–glyoxylate aminotransferase	47.7	27	Rosetta 2-DE3, 15°C	N.D.
KES20403, glutamate-1-semialdehyde 2,1-aminomutase	45.4	26	Failed	
KES23973, adenosylmethionine-8-amino-7-oxononanoate aminotransferase	54.2	25	BL21-DE3, 37°C	N.D.
KES24511, aminotransferase	50.2	23	Rosetta 2-DE3, 15°C	Putrescine
KES23458, beta-alanine–pyruvate aminotransferase	48.1	19	BL21-DE3, 37°C	β-alanine
KES21511, glutamate-1-semialdehyde aminotransferase	44.3	18	Rosetta 2-DE3, 15°C	3-Aminocyclohexanoate
KES22518, hypothetical protein	42.2	4	Failed	

a Accession numbers corresponding to GenBank assembly.

b Putative assignments for each protein predicted by BLAST analysis in UniProt database.

c Protein size (native, untagged peptide sequence). % identity to GabT from *E. coli* BL21-DE3 (scoring was completed using Clustalo) is detailed. Optimal experimental conditions for protein overexpression in *E. coli* and the substrate with which the greatest activity for each protein was observed are also reported.

N.D., activity not determined.

Analysis of the genomic context for each of the putative TA genes failed to identify which of the genes, if any, might encode a 12-aminododecanoic acid TA. However, the analysis did suggest possible physiological roles for some of the encoded enzymes (Fig. S1).

**Fig 2:**
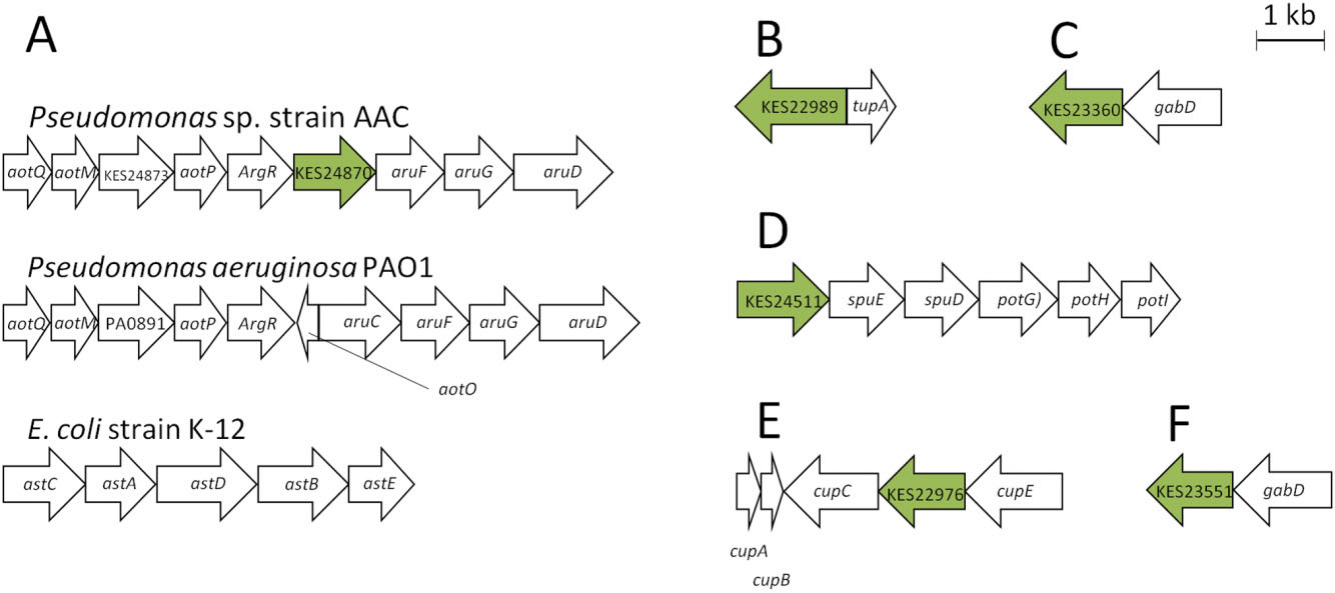
Putative operons associated with transaminases identified in this study. A) Comparison of the genes involved in the metabolism of arginine for *Pseudomonas* sp. strain AAC, *Pseudomonas aeruginosa* PA01 and *E. coli* strain K-12. The transaminase of interest is shown in green. AotQ – Arginine/ornithine transport protein; AotM – Arginine/ornithine transport protein; AotP – Arginine/ornithine transport protein; ArgR -Transcriptional regulator; KES24870 / AruC– acetylornithine aminotransferase; AruF – Arginine/ornithine succinyltransferase alpha subunit; AruG – Arginine/ornithine succinyltransferase beta/all subunit; AruD – N-succinylglutamate-5-semialdehyde dehydrogenase; AstC – Succinylornithine transaminase; AstA – Arginine-N-succinyltransferase; AstD – N-succinylglutamate-5-semialdehyde dehydrogenase; AstB – N-succinylarginine dihydrolase; AstE – Succinylglutamate desuccinylase. B) The mobile element containing taurine-utilization proteins. KES22989 – Taurine–pyruvate aminotransferase; TupA – ABC Taurine transporter. C) Genes involved in pyruvate-dependent 4-aminobutyrate metabolism. KES23360 – Uncharacterized protein; GabD – Succinate semialdehyde dehydrogenase. D) Genes involved in polyamine (putrescine) transport production. KES24511 – Putrescine aminotransferase; SpuE – Polyamine transport protein; SpuD – Putrescine ABC transport protein; PotG – Polyamine transport protein; PotH – Polyamine transport protein; PotI – Polyamine transport protein. E) A second polyamine (cadaverine) transport domain, responsible for the utilization of cadaverine. CupA – Putative polyamine export protein; CupB – Putative polyamine export protein; CupC – Putative cadaverine utilization protein; KES22976 – 4-aminobutyrate aminotransferase; CupE – Putative dehydrogenase. F) GabT homologue and accompanying dehydrogenase, GabD, within *Pseudomonas* sp. strain AAC. KES23551 – 4-aminobutyrate aminotransferase; GabD – Succinate-semialdehyde dehydrogenase.

KES24870 appears to be a succinyl/acetyl ornithine TA (Newman *et al*., 2013), an enzyme typically involved in the catabolism and/or anabolism of the amino acid arginine. The genes surrounding the TA-encoding gene support this function (Fig. S1; Fig. 2A). The arrangement of the genes in this area of the genome is identical to that of *Pseudomonas aeruginosa*, with the region containing the *aot* (Nishijyo *et al*., 1998) (arginine/ornithine transporter) genes, the *aru* (Itoh, 1997) (arginine utilization) genes and the gene encoding the arginine-responsive transcriptional regulator, ArgR. It is also analogous to the region of the *E. coli* genome that contains the *ast* genes responsible for arginine catabolism (Schneider *et al*., 1998). Moreover, when overexpressed in *E. coli*, the enzyme encoded by KES24870 was able to catalyze the amine-transfer reaction between *N*-acetyl-l-ornithine and α-ketoglutarate (specific activity 1.2 μmol min^−1^ mg^−1^).

KES23973 has homology with adenosylmethionine-8-amino-7-oxononanoate TA (diaminopelargonic acid synthetise; *bioA*), which is involved in a step of biotin synthesis in which S-adenosyl-l-methionine is deaminated to produce *S*-adenosyl-4-methylthio-2-oxobutanoate using 8-amino-7-oxonononanate as an amine acceptor. While the genes encoding biotin synthesis are co-located in a single operon in *E. coli* (Streit and Entcheva, 2003), KES23973 does not appear to be associated with a similar operon (Fig. S1). Moreover, in the *P*. *aeruginosa* PAO1 genome, the *bioA* homologue is not found in association with any other biotin synthesis genes (Stover *et al*., 2000). Unfortunately, the co-substrate for this reaction (8-amino-7-oxonononanate) is not readily available, and so we have not tested the activity of the enzyme encoded by this gene. However, in the absence of an alternative *bioA* homologue, we would tentatively assign the function of this enzyme as that of BioA in biotin synthesis.

KES22989 has homology with taurine : pyruvate aminotransferases. Moreover, it is found in proximity to a gene encoding a taurine transporter (Fig. S1; Fig. 2B). It is likely that this two gene cluster is a taurine-utilization operon; however, we were not able to test the specificity of the TA because of difficulties expressing the protein (despite extensive efforts). In addition, there is also a nearby integrase, which may suggest that this operon is contained in a mobile element.

KES21039 was predicted to be a diaminobutyrate/α-ketoglutarate TA, associated with amino acid (glycine, threonine and serine) and diaminopropane anabolism (Ikai and Yamamoto, 1997), as well as the synthesis of the compatible solute ectoine in halophiles (Kuhlmann and Bremer, 2002). The genomic context of this gene reveals little about its physiological role (Fig. S1), and indeed we found that the enzyme did not exhibit its predicted activity. However, the protein was found to have some TA activity with two substrates, β-alanine and aminoisobutyrate (pyruvate co-substrate, specific activity 4.4 nmol min^−1^ mg^−1^ for each). The observed activities were indistinguishable, and very low, and as such they may be promiscuous functions for the enzyme. KES23458 was predicted to be a β-alanine/pyruvate TA (Hayaishi *et al*., 1961) and was shown to possess this activity *in vitro* (specific activity 38.7 nmol min^−1^ mg^−1^), although there is little additional evidence beyond the homology of KES23458 with β-alanine/pyruvate TAs that would indicate this activity (Fig. S1).

Gene KES23360 was not assigned a putative function, but was most closely related to a characterized diaminobutyrate TA from *Halomonas elongata* (63% identity, GenBank accession number: CBV43545). However, it is adjacent to a gene with homology to succinate semialdehyde dehydrogenases in the *Pseudomonas* sp. strain AAC genome (Fig. S1; Fig. 2C). As this arrangement is common for 4-aminobutyrate degradation operons in other genomes (Stover *et al*., 2000), it is likely that KES23360 is a GabT homologue. 4-Aminobutyrate activity was observed when pyruvate was used as a co-substrate (specific activity 169 nmol min^−1^ mg^−1^), and as such this enzyme may be involved in an alternative 4-aminobutyrate metabolic pathway similar to that reported for *Rhizobium* sp. (Prell *et al*., 2009) and can therefore be assigned the function of omega amino acid TA.

KES24511 appears to be involved in polyamine metabolism, and it has greatest homology to putrescine aminotransferases (90% identity to NP_248990; Table 1). Overexpression of this protein in *E. coli* was accompanied by the aroma of polyamines, and the enzyme itself had specificity for putrescine (specific activity 134.2 nmol min^−1^ mg^−1^). The region of the genome surrounding KES24511 contained a number of genes that have homology with polyamine metabolism-related genes (Fig. S1; Fig. 2D), and it therefore seems likely that KES24511 encodes a putrescine TA.

KES22976 was predicted to be a 4-aminobutyrate aminotransferase, albeit this gene is co-located with spermidine transporters, a 4-aminobutyrate permease and a succinate-semialdehyde dehydrogenase (Fig. S1; Fig. 2E). When tested, the enzyme encoded by KES22976 had no 4-aminobutyrate aminotransferase activity, nor activity against spermidine. However, it was found to have activity against the related polyamine, cadaverine, with pyruvate as co-substrate (specific activity 12.6 nmol min^−1^ mg^−1^). This suggests that this region of the *Pseudomonas* sp. strain AAC genome may encode cadaverine-utilization proteins.

Neither proteins encoded by genes KES22518 and KES25161 could be assigned a putative function by BLAST analysis, and the genomic context of the two genes revealed no obvious clues as to their respective functions (Fig. S1). The protein encoded by KES22518 could not be produced in a soluble form despite extensive efforts and therefore remains uncharacterized. However, KES25161 produced soluble protein and was therefore characterized using a broader substrate screen. Activity was demonstrated with two substrates, 3-aminocyclohexanoic acid (specific activity 1.6 nmol min^−1^ mg^−1^) and 3-aminoheptanoic acid (specific activity 75.8 nmol min^−1^ mg^−1^; both using α-ketoglutarate as co-substrate), both of which are β-amino acids. As such we would tentatively assign a function of β-amino acid TA to KES25161.

Both KES21511 and KES20403 were predicted to be genes encoding a glutamate-1-semialdehyde-2,1-aminomutase, known to participate in porphyrin synthesis (Johansson and Hederstedt, 1999). There were no obvious indications for the physiological function of either gene product from their genomic context (Fig. S1), and we were unable to source a suitable substrate to test for aminomutase activity. A broader screen for activity did, however, highlight that KES21511 was able to catalyze the transamination reaction between 3-aminocyclohexanoate and α-ketoglutarate (specific activity 15.1 nmol min^−1^ mg^−1^). This activity suggests that the protein has at least folded correctly, but provides little further information on its physiological role.

KES21385 was predicted to be an alanine/glyoxylate TA, although there is little supporting evidence for this from its surrounding genes (Fig. S1). The protein was expressed in soluble form and tested for the predicted activity, but no reaction was observed. A broader range of substrates was tested, but no activity could be determined for this protein. As such KES21385 remains uncharacterized.

The protein encoded by KES23551 had up to 96% identity with 4-aminobutyrate aminotransferases and was found in proximity to a succinate semialdehyde dehydrogenase homologue (Fig. S1; Fig. 2F). When expressed in *E. coli*, the KES23551 enzyme possessed 4-aminobutyrate/α-ketoglutarate aminotransferase activity (specific activity 430.5 nmol min^−1^ mg^−1^), and we would suggest that this gene encodes the functional GabT homologue for this bacterium.

Note that in each case, specific activity was measured using a dehydrogenase-coupled assay. The limitation of this assay is that high levels of co-substrate could be supplied to the TA, as they inhibit dehydrogenase-coupled reactions (Newman *et al*., 2013). As a result, although a constant supply of co-substrate was maintained, substrate concentration is significantly higher than co-substrate concentration, and the reactions were likely to have been suboptimal for TA activity. The advantage of the assay, however, is its high sensitivity, allowing for poor substrates to be identified with reasonable throughput.

### ω-Aminoalkanoate TA activity was discovered in several *Pseudomonas* sp. strain AAC TAs

**Fig 3:**
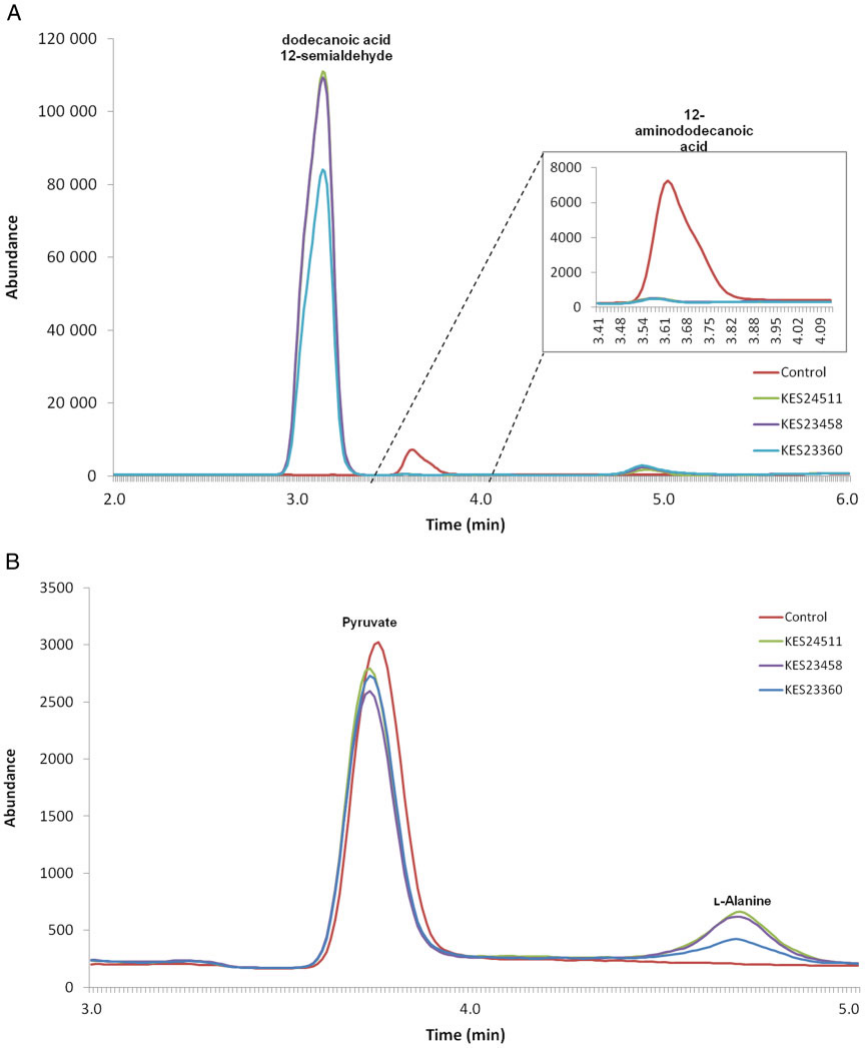
Transaminase-catalyzed transformation of 12-aminododecanoic acid.A. LC-MS trace showing complete conversion of 12-aminododecanoic acid material into dodecanoic acid-12-semialdehyde by KES24511, KES23458 and KES23360. A negative control (no transaminase enzyme) is also shown. The inset magnifies the 12-aminododecanoic acid, which is far less UV-absorbant than dodecanoic acid-12-semialdehyde.B. LC-MS trace showing alanine (co-product) formation *via* 12-aminododecanoic acid-dependent amination of pyruvate by KES24511, KES23458 and KES23360. Peaks corresponding to pyruvate and l-alanine were verified against pure standards.

As noted above, 11 of the 14 TAs identified in *Pseudomonas* sp. strain AAC were expressed in *E. coli*. This allowed each of the 11 to be screened for activity against 12-aminododecanoic acid using dehydrogenase-coupled assays and a saturated solution of 12-aminododecanoic acid. Using common TA co-substrates pyruvate and α-ketoglutarate, two dehydrogenase assays were carried out for each TA, a pyruvate/alanine dehydrogenase and an α-ketoglutarate/glutamate dehydrogenase (GDH)-coupled assay. Each assay was conducted at pH 9 and monitored at 340 nm as previously described. Hyperbolic changes in absorbance corresponding to NADH formation, and ultimately 12-aminododecanoic acid metabolism, were observed with three enzymes, KES24511, KES23458 and KES23360. To confirm the results of the UV assay, reactions were also analysed by LC-MS. Analysis of the resulting spectra identified alanine, the co-product of the TA reaction, as well as a new peak with *m/z* = 213, which corresponds to the −1 ion of the expected semialdehyde product. In addition, no residual starting material could be detected (see LC-MS traces, Fig. 3).

Additional work to better understand the 12-aminododecanoic acid : pyruvate TAs, encoded by genes KES24511, KES23458 and KES23360, is now underway. It is anticipated that these could become viable biocatalysts for bionylon monomer production.

## Experimental procedures

### Materials

Target genes were amplified using Phusion® DNA polymerase (Thermo). Genes were ligated into the pETcc2 (Peat *et al*., 2013) (modified pET14b, Novagen) expression vector using *Nde*I and *Bam*HI restriction endonucleases and T4 DNA ligase (New England Biolabs). All proteins were expressed and purified from either *E. coli* BL21 (DE3), purchased from Novagen, or in *E. coli* Rosetta 2 (DE3) cells, purchased from EMD Millipore. All chemicals were ordered from Sigma Aldrich (St Louis, MO, USA). Synthetic genes were purchased from GeneART (Invitrogen).

### Isolation and identification of 12-aminododecanoic acid-utilizing bacterium

Bacterial cultures were grown in McCartney bottles at 28°C overnight in 20 mL Luria–Bertani (LB) medium (Bertani, 1951). The cultures were sedimented by centrifugation in a Beckman GS-6R bench top centrifuge at 4000 r.p.m. for 20 min at 4°C. The cell pellets were then re-suspended in 20 mL M9 medium (Cold Spring Harbor Protocols, 2006) to which no nitrogen source had been added. The cells were rinsed in N-free M9 medium three times before being suspended in 20 mL N-free M9 medium. Inocula of 100 μL were used to seed 20 mL cultures of N-free M9 medium that had been supplemented with 200 mg of 12-aminododecanoic acid, which were then grown at 28°C for up to 1 month. Where growth was observed, cultures were streaked to single colonies on M9 agar (15%, w/v) supplemented with 10 mg mL^−1^ 12-aminododecanoic acid.

Genomic DNA from these cultures was prepared using a Macherey-Nagel NucleoSpin® Tissue kit following the manufacturer's instructions. 16S rDNA was amplified using 27f (5′ AGAGTTTGATCMTGGCTCAG 3′) and 1492r (5′ TACGGYTACCTTGTTACGACTT 3′) primers and Expand DNA polymerase (Roche). 16S rDNA amplicons were cloned into pCR-II TOPO, using the TA cloning kit (Invitrogen, Australia) and sequenced by the Micromon DNA sequencing facility (Melbourne, Vic., Australia). The complete genome of the 12-aminododecanoic acid-utilizing bacterium was sequenced by the BGI (China).

### Bioinformatics

Target TAs from the genome of the 12-aminododecanoic acid-utilizing bacterium were identified using Exonerate protein2genome sequence alignment (default settings). 4-Aminobutyrate TA (GabT) was used as the query sequence against the genome (target). From the partial alignment output, the relevant genes were identified, and primers designed for cloning the genes from genomic DNA. Gene identities were calculated versus GabT from *E. coli* BL21 λDE3 using Clustal Omega in UniProt (The UniProt Consortium, 2014), and putative functional assignments were made based on the annotated functions of the genes closest to each TA following a BLAST of each gene on UniProt.

### Cloning TA-encoding genes

Each of the gene fragments was amplified by PCR from genomic DNA using primers detailed in Table S1. Amplicons were digested using *Nde*I and *Bam*HI restriction endonucleases (NEB) and ligated (T4 DNA ligase, NEB) into pETcc2. Ligations were used to transform electrocompetent *E. coli* BL21 λDE3 and plated on LB agar plates containing ampicillin (100 μg mL^−1^). Following incubation at 37°C overnight, transformants were used to inoculate 10 mL LBAmp cultures. Plasmid DNA was isolated from the cultures using a Qiagen Miniprep Kit, according to the manufacturers' instructions and sequenced by Macrogen (South Korea) to confirm the construct. *Escherichia coli* codon-optimized synthetic genes for TAs KES22518, KES22989 and KES20403 were provided in the pMA vector (Mammalian Gene Collection, 2014). Sequences are detailed in Appendix S1. These three synthetic genes were subcloned into pETcc2 using *Nde*I and *Bam*HI, and ligated with T4 DNA ligase. Constructs were verified by DNA sequencing.

### TA expression and purification

Protein overexpression was achieved by growing 200 mL of *E. coli* BL21 λDE3 containing the desired construct in LB media containing ampicillin (100 μg mL^−1^) at 37°C. When the OD_600_ reached 0.6–1.0, the cultures were induced by the addition of isopropyl β-D-1-thiogalactopyranoside (1 mM final concentration) and further incubated at either 15°C or 37°C for 18 h. The cells were isolated by centrifugation (4500 r.p.m.; 20 min) and the supernatant discarded. Pellets were re-suspended in potassium phosphate buffer (10 mM, pH 7.5), and cell lysis was achieved using BugBuster® 10 × reagent (Merck) with shaking on ice for 1 h. Cellular debris was precipitated by centrifugation (18 000 r.p.m., 45 min). and cell-free extracts were passed over a HiTrap Chelating HP column (GE Healthcare) on an Åkta FPLC (Fast Protein Liquid Chromatography, GE Healthcare) against an increasing concentration of imidazole (5–500 mM). Eluted proteins were washed in phosphate buffer (10 mM, pH 7.5) and concentrated by centrifugation utilizing spin columns (GE Healthcare; 10 kDa MWCO). Purity was assessed by SDS-PAGE (shown in Fig. S2). Five of the TAs (genes KES24511, KES21511, KES21385, KES21039 and KES22976) did not express soluble protein under these conditions. To address this, the constructs were re-transformed into *E. coli* Rosetta2 λDE3 cells (Merck Millipore) and expressed in LB media containing ampicillin and chloramphenicol (100 μg mL^−1^ and 35 μg mL^−1^ respectively). All other aspects of the protocol remained unchanged.

### Determination of substrate specificity

**Fig 4:**
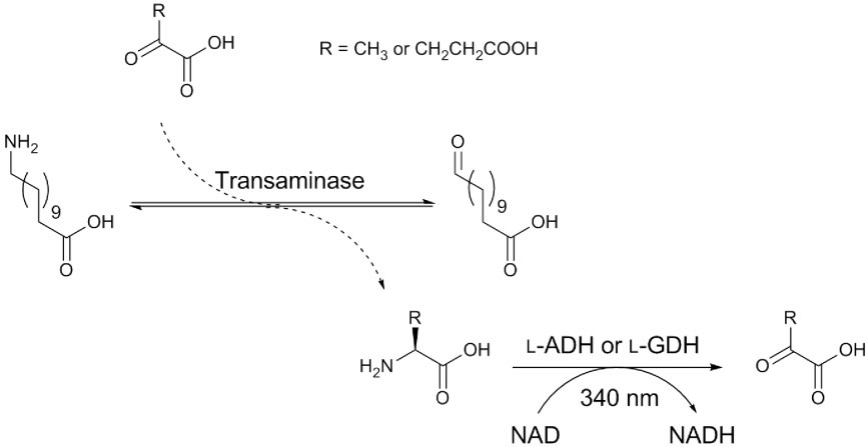
Dehydrogenase-coupled UV assay for transaminase activity. The transaminase catalyzes the transfer of the amine from the 12-aminododecanoic acid to the α-keto acid co-substrate, producing the C12 semialdehyde and alanine or glutamate as a co-product. The dehydrogenase then catalyzes the oxidative deamination of the co-product utilizing a cofactor, NAD (nicotinamide adenine dinucleotide), which is concurrently reduced to NADH. Formation of NADH can be detected by UV photospectrometry as a hyperchromic shift at 340 nm.

The rates of deamination of amines by each of the TAs was assessed using enzyme-coupled dehydrogenase assays as outlined in Fig. 4. A typical assay comprised: 6.25 mM substrate, co-substrate (0.5 mM pyruvate or 0.25 mM α-ketoglutarate), 1.25 mM NAD^+^, dehydrogenase (0.035 U of alanine dehydrogenase or 0.925 U of GDH), 0.025–2 μM TA and 100 mM potassium phosphate (pH 9). In the cases of KES23973 and KES21385, where no activity was determined, the pH of the phosphate buffer was varied from 7.5 to 10 to further scope out enzyme activity, although no activity was observed with any substrate in the range tested. The rates of the TAs were inferred from the coupled rate of NAD^+^ turnover by the amino dehydrogenase, which was dependent on the production co-product (alanine or glutamate) by the TA. NAD^+^ turnover was measured by the change in UV absorbance at 340 nm using a SpectraMax M2 spectrophotometer (Molecular Devices, Australia); reactions were conducted at 28°C at pH 9 unless otherwise specified.

### Mass spectrometry

To corroborate the findings of the UV assay, similar assays were carried out and analysed by LC-MS (Agilent 1260 Infinity with a 6120 Quadrupole mass analyzer). Fifty millilitres of 12-aminododecanoic acid (1 mg mL^−1^ solution) and 10 μL of pyruvate (50 mM stock) were added to TA enzymes (KES24511, KES23458 and KES23360; between 50 and 1400 pmol protein) in potassium phosphate buffer (100 mM, pH 10). The reaction was mixed for 3 h before LC-MS analysis. LC was carried out using an Astec Chirobiotic T column (5 μm × 25 cm × 4.6 mm) and a mobile phase of methanol/water/formic acid (70:30:0.02 v/v) at 1 mL min^−1^.

## Supporting Information

**Fig. S1.** Illustration of the genes surrounding the transaminases in the *Pseudomonas* sp. strain AAC genome. The transaminases of interest are shown in green. The NCBI accession numbers for each protein and the predicted functions of each following BLAST analysis are detailed, along with the % identity and e values in parentheses. **Fig. S2.** SDS-PAGE analysis of each of the successfully expressed proteins. Left: cell-free extracts for all proteins. Middle: each protein following purification by affinity chromatography. Right: KES24870 following purification by affinity chromatography. **Table S1.** Primers for subcloning of the 14 target transaminases into pETcc2. Restriction sites are underlined. **Appendix S1.** Synthetic gene sequences for KES22518, KES22989 and KES20403. *Nde*I and *Bam*HI restriction sites are underlined.
