# Supplementary material for: Identification of novel transaminases from a 12-aminododecanoic acid-metabolizing Pseudomonas strain
Source: Microb Biotechnol. 2015 Apr 24;8(4):665–72. doi: 10.1111/1751-7915.12278 (PMC4476821; doi:10.1111/1751-7915.12278)
Supplement: Supplementary file 1 — Fig. S1. Illustration of the genes surrounding the transaminases in the Pseudomonas sp. strain AAC genome. The transaminases of interest are shown in green. The NCBI accession numbers for each protein and the predicted functions of each following BLAST analysis are detailed, along with the % identity and e values in parentheses. Fig. S2. SDS-PAGE analysis of each of the successfully expressed proteins. Left: cell-free extracts for all proteins. Middle: each protein following purification by affinity chromatography. Right: KES24870 following purification by affinity chromatography. Table S1. Primers for subcloning of the 14 target transaminases into pETcc2. Restriction sites are underlined. Appendix S1. Synthetic gene sequences for KES22518, KES22989 and KES20403. NdeI and BamHI restriction sites are underlined. [file mbt20008-0665-sd1.docx]

**Identification of Novel Transaminases from a 12-Aminododecanoic Acid-metabolizing *Pseudomonas* strain.**

Matthew Wilding^1,2^, Ellen F. A. Walsh^3^, Susan J. Dorrian^1^ and Colin Scott^1^

*^1^CSIRO Land and Water Flagship, Black Mountain, Canberra, Australia, 2601*

*^2^CSIRO Food and Nutrition Flagship, Black Mountain, Canberra, Australia, 2601*

^3^*Research School of Chemistry, Australian National University, Canberra, Australia, 2601*

**Supplementary Information**


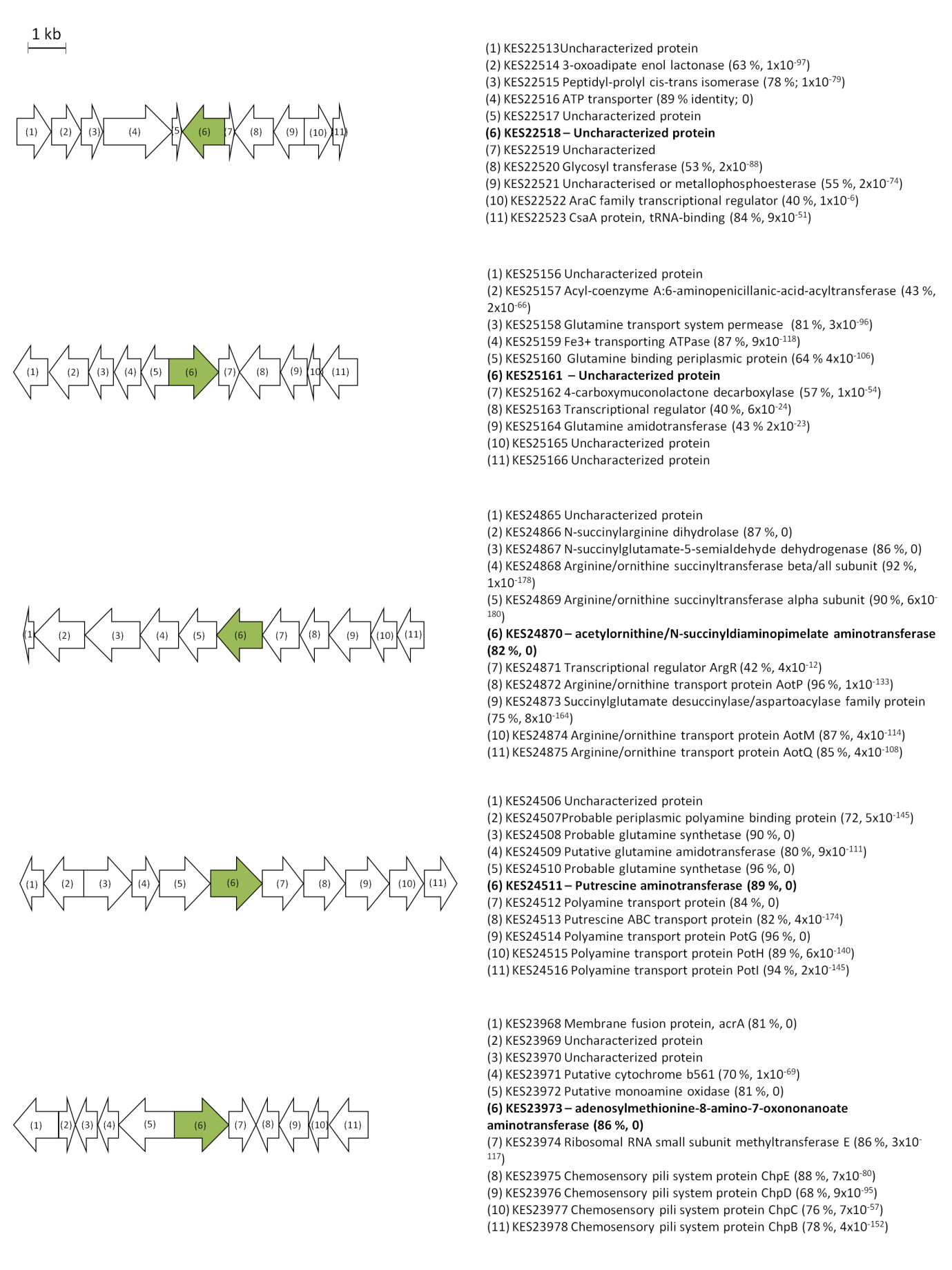


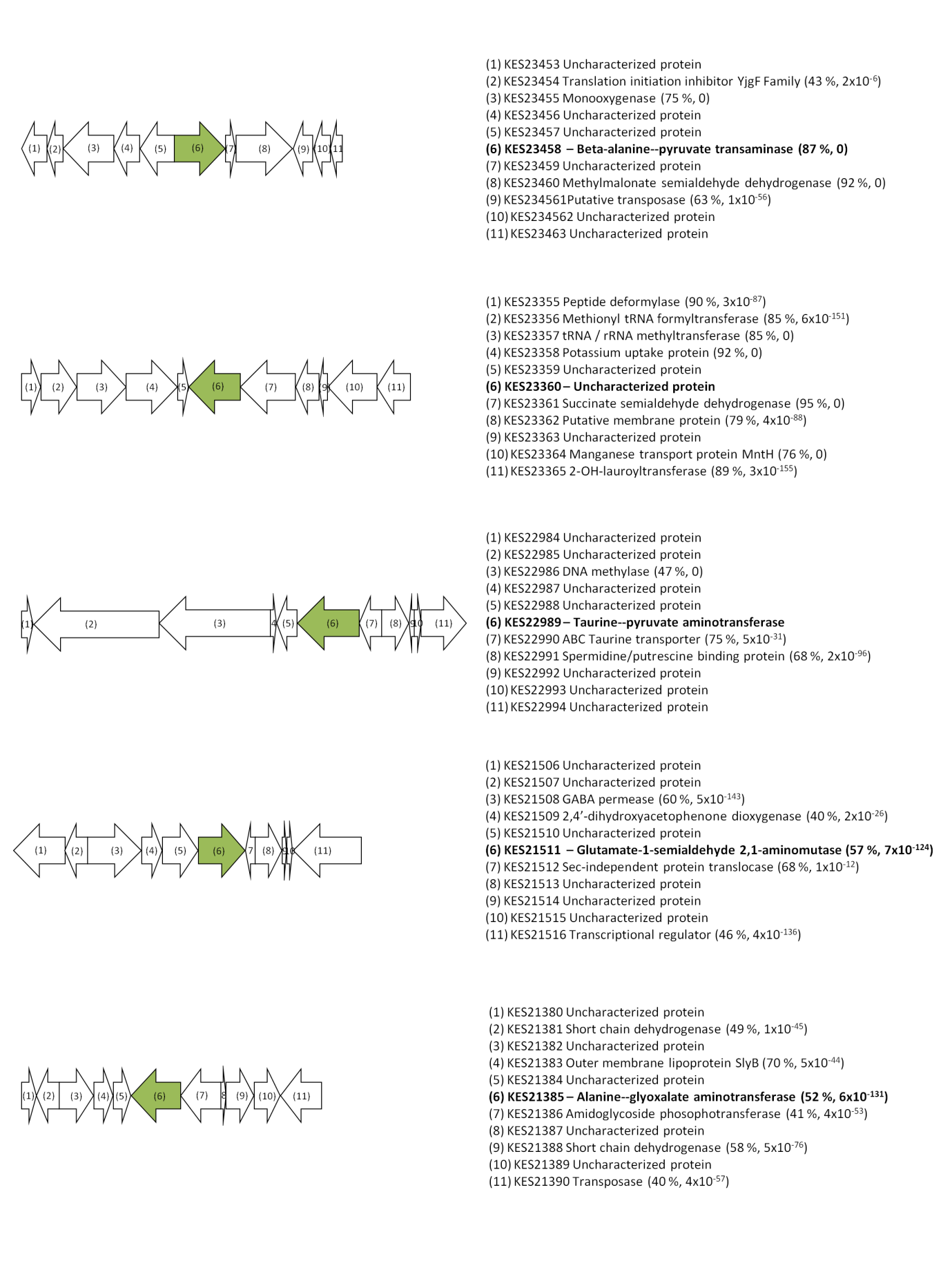


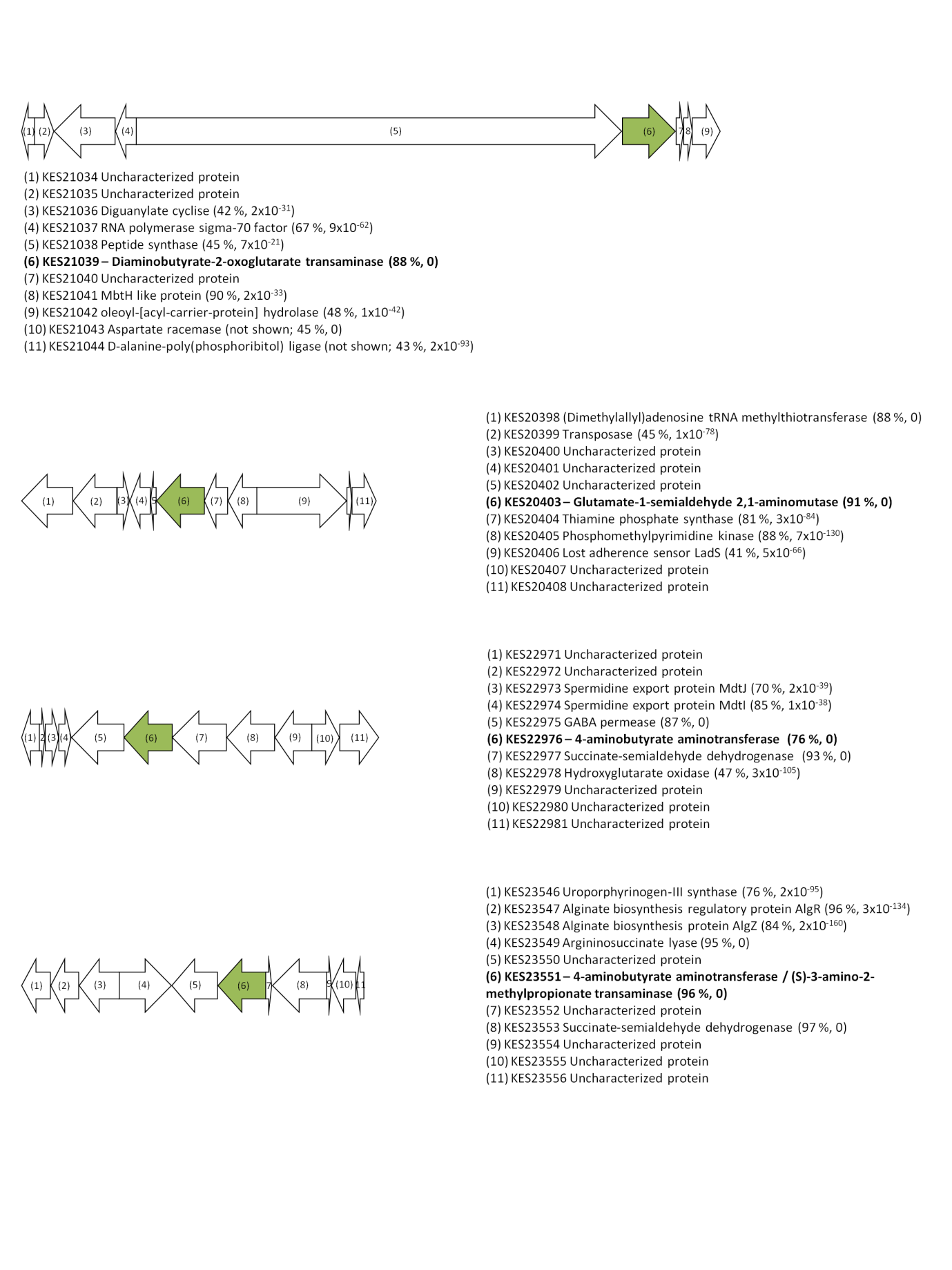


Supplementary Figure 1 – Illustration of the genes surrounding the transaminases in the *Pseudomonas* sp. strain AAC genome. The transaminases of interest are shown in green. The NCBI accession numbers for each protein and the predicted functions of each following BLAST analysis are detailed, along with the % identity and e values in parentheses.

| Primer | Sequence (5' to 3')^a^ | |
| --- | --- | --- |
| KES22518 Fwd | TGTCCTTCATATGCCTATCCACAGACTTTCCCGCCTCGGC | |
| KES22518 Rev | AAAAAAGGATCCTTACTCCGCCTGCCGGTAAGGCAGCGCC | |
| KES25161 Fwd | TTTTTTCATATGAGCCACGTCCTGCACCGCAGCCTCAC | |
| KES25161 Rev | AAAAAAGGATCCTCAGAGGCTGCCGATGGCCTGGTCGATG | |
| KES24870 Fwd | TTTTTTCATATGTCCGCTCCGCTCGCTCAGGTACAACGCTC | |
| KES24870 Rev | AAAAAAGGATCCTCAGCCGCGGGTGAGCTTGGCCACC | |
| KES24511 Fwd | TTTTTTCATATGACGAAACAGACCAGCGCCCAGACCCAAC | |
| KES24511 Rev | AAAAAAGGATCCTCAAGACAGCGCTACGGCGGCGGTTTG | |
| KES23973 Fwd | TTTTTTCATATGTCCCATTGCATAGGCTTGCCCGACGAC | |
| KES23973 Rev | AAAAAAGGATCCCTAGCCCGGGTCCCGATGATCCGGGTAC | |
| KES23458 Fwd | TTTTTTCATATGAACCAGCAAGTGAACGTAGCGCCGTC | |
| KES23458 Rev | AAAAAAGGATCCTTACGCCACCCCGTTGAGGGCTTC | |
| KES23360 Fwd | TTTTTTCATATGACCGACTACGCCAAGCTGTTCGAGCAGG | |
| KES23360 Rev | AAAAAAGGATCCTTAAAGCACCTCCGCCGCCACCGCGTCC | |
| KES22989 Fwd | TTTTTTCATATGAACGATTTTTCCCCGCAGTCCGCCAG | |
| KES22989 Rev | AAAAAAGGATCCTTATTCCTTTCCCTGCCCGCACACGGGAAG | |
| KES21511 Fwd | TTTTTTCATATGGCCCATCTGGTGGGTGACGTATCCAG | |
| KES21511 Rev | AAAAAAGGATCCTTACAAGAGCACCTCGGCTAAACGCTCC | |
| KES21385 Fwd | TTTTTTCATATGACCATGACCAATGGCTACAGCCCGGCCGAC | |
| KES21385 Rev | AAAAAAGGATCCTTAGCCGGCGGTTTCGGCCAGGGC | |
| KES21039 Fwd | TTTTTTCATATGCAAGTCGCTACCAGCGTCATCGATGACC | |
| KES21039 Rev | AAAAAAGGATCCTTACAATTTGCCGGCAGGCACCGCCGC | |
| KES20403 Fwd | TTTTTTCATATGTCCCGTTCCGAACTCCTCTTCGCCAAC | |
| KES20403 Rev | AAAAAAGGATCCTTACTTGAGGGCGGCGAAGGCGCGCTCG | |
| KES22976 Fwd | TTTTTTCATATGAACAGCAACCAGAGCCTGCAAGAACGGCGCATGGC | |
| KES22976 Rev | AAAAAAGGATCCTCAGGCCAGCTCGTCGAAGCACTCGCCGATGATG | |
| KES23551 Fwd | TTTTTTCATATGAGCAAAACCAACGAATCCCTGCTGCAACGCCGCCAG | |
| KES23551 Rev | AAAAAAGGATCCTCAGGCCAGTTCGTCGAAGCACTCGGCCAGGATG | |
| ^a^Restriction sites underlined | |  |

Supplementary Table 1 – Primers for sub-cloning of the fourteen target transaminases into pETcc2. Restriction sites are underlined.


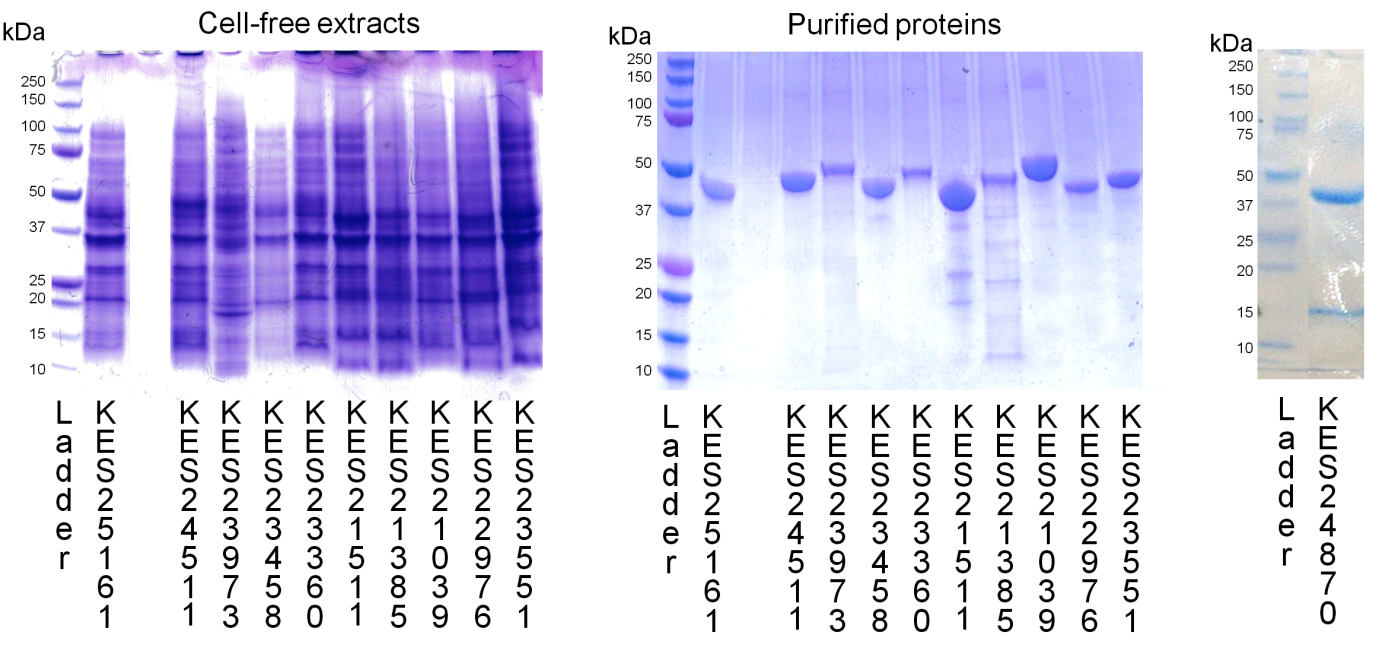


Supplementary Figure 2 – SDS-PAGE analysis of each of the successfully expressed proteins. Left: Cell-free extracts for all proteins. Middle: Each protein following purification by affinity chromatography. Right: KES24870 following purification by affinity chromatography.

Synthetic gene sequences for KES22518, KES22989 and KES20403. *Nde*I and *Bam*HI restriction sites are underlined.

KES22518:

CATATGCCGATTCATCGTCTGAGCCGTCTGGGTGATCTGGCACCGAGCCAGTGGGATGCACTGCTGCCGGATGCACAGCCGTTTCTGCGTCATGCATTTCTGGCAAGCCTGGAAGATAGCGGTAGCGTTACCGCACGTACCGGTTGGCGTCCGGCACATCATGTTCTGCTGGATGATGCAGGTCGTCTGCGTGCAGCACTGCCTGCCTATGTTAAAGCACATAGTTATGGCGAATATGTGTTTGATTGGGGTTGGGCAGATGCATGTCAGCGTGCAGGTATTCGTTATTATCCGAAACTGCTGGTTGGTGTTCCGTTTAGTCCGGTTAGCGGTGCACGTCTGCTGGGCACCGATGAAGGTATTGCAGAACTGCTGGGTGTGCTGGCCGAAGGCACAGCCGAAGGTGTTTGGAGCGGTGTTCATGTGAATTTTACCGATCCGCGTGCAGATGGTCTGATTGCAGCACAGGGTGGTTGGCTGGAACGTCTGGGTTGTCAGTATCATTGGTTTAATCGTGGTTATCGCGATTTCCAGGATTTTCTGGATGCCCTGGCCAGCCGTAAACGTAAACAGCTGCGTAAAGAACGTGAACAGGTTGCAGGTCAGGGTATGGAATTTCGTTGGATGGAAGGTCCGGAACTGGCAGAAGCAGATTGGGATTTTGTTTATGCATGTTATGCCAGCACCTATGAAGTTCGTGGTCAGGCACCGTATCTGACCCGTGAATTTTTCAGCCTGCTGGCAGAACGTATGGGTGCAGCAATTCGTGTTGTTTTTGCACGTCGTAATGGTCGTCCGGTTGCAATGGCATTTAGCCTGGTTGATGGTGATACCCTGTATGGTCGTTATTGGGGCTGTCTGGCAGAATATGATCGTCTGCATTTTGAAACCTGCTTTTATCAGGGTCTGGATTATGCACTGGCAGCAGGTCTGCGTCGTTTTGATGCGGGTGCCCAGGGTGAACATAAACTGATTCGTGGTTTTGAACCGGTGCTGACCCGTAGCTGGCATCAGCTGGGTCATCCGGGACTGCGTGCCGCAGTTGATGATTTCCTGGCACAAGAACGCGCAGGCATTCGTGCCTATGCAGAAGAAGCACGCGCAGCCCTGCCGTATCGTCAGGCAGAATAAGGATCC

KES22989:

CATATGAACGATTTTAGTCCGCAGAGCGCAAGCGAACAGTGGCTGGAAAGCGATCGTCGTCATGTTTGGCATCATCTGACCCAGCATCGTGGTTTTGAACGTGCAGCACCGCCTATGATGCATCGTGGCGAAGGTCTGTATGTTTGGGATGTTCAGGGTCGTCGTTATCTGGATGCAACCAGCGGTGGTGTTTGGTGTGTTAATGTTGGTTATGGTCGTGCAAGCATTGCAGATGCAGTTCGTGATCAGCTGGTTGAACTGAACTATTTTGCAGGCACCCTGGGTAGTCCGGTTGCAGCACGTTTTGCAGAAGCACTGATTGAAAATATGCCTGGTATGAGCCGTGTGTATTTTAGCAATAGCGGTAGCGAAGCAAACGAGAAAGCCTATAAAATGGTTCGTCAGATTGCCCATCGTAAATATGCAGGTCGCAAACACAAAATTCTGTATCGTGAACGTGATTATCATGGCACCACCATTACCTGTCTGAGCAGCACCGGTCAGCAAGAACGTCGTATGCAGTATAGCCCGTTTACACCGGGTTTTGTTGAATTTCCGCATTGTTGTGAATATCGCAGCCAGTTTGGTCCGGTTGATGATTATGGTGCACGCGCAGCACGTGCAATGGAAGAAGTTATTCTGCGCGAAGGTCCGGATACCATTGGTGCAGTTGTTCTGGAACCGATTACCGCAGGCGGTGGCGTTATTACCCAGCCTGCAGGTTATCTGAAAGCAGTTGAAGCAATTTGCCGCAAATATGATATCCTGATCCATATCGATGAAGTTGTTTGTGGTCTGGGTCGTACCGGTAAATGGTTTGCATATCAGCATTATGGTATTCAGCCGGATATTGTTACCATGGCAAAAGGTGTTGCAAGCGGTTATGCAGCAATTAGCTGTACCGTTACCACCGAAGAAGTTTTTGAACAGTTTAAAGGTGAACCGGATGATCGCGAAGTTTATTTTCGTGATATTAGCACCTTTGGTGGTTGTACCGCAGGTCCGGCAGCAGCACTGGAAAATCTGCGTATTATTGAACGTGAAGGTCTGCTGGAAAACGCCGAAAAAATTGGTGCATACCTGTTTGAAGGCCTGCTGCGTCTGCAGCGTAAACATCCGCTGATTGGTGAAGTTCGTGGTAAAGGTCTGTTTCTGGGTATTGAACTGGTTAGCGATCGTGAAACCCGTGAACCGGTTGCCGAAGGTGTGGCAATGCGTGTTGCAGCCCTGTGTATGGCCAAAGGTCTGATTATTAGCCGTACCAATCGTAGCTTTGCCGATTTTAACAATACCCTGTGTCTGAGTCCGGCACTGACCATTGGTCGTGAACAGGCAGATCAGATTCTGAGCATTCTGGAACAGGCACTGGCCGAAGTTGGTGAAGCCAGCGAAGAAAGCAATCGTACAGCCGGTGTTAGCGAAGAACTGGCACCGGCATGGTTTCGTCCGGGTGAAGCAGATGATGATCGTCATCGTGCAAATGCCGGTGAAGAAAGCTCAAGCAGCCGTGAAACCGGTGGTACATTTCGTGGTCGTCGTGATGGTGATGGTCTGAGCGAAGCAGTGGGTTATGAACATACCAGCGATCTGGTTGCCTCACATGGTAAAACCCCGACCCTGTTTCTGCCGGTTTGCGGTCAGGGTAAAGAATAAGGATCC

KES20403:

CATATGAGCCGTAGCGAACTGCTGTTTGCAAATGCACAGAAACATATTCCGGGTGGTGTTAATAGTCCGGTTCGTGCATTTAAAAGCGTTGGTGGTACACCGCTGTTTTTCAAACATGCCGAAGGTGCCTATGTTGTGGATGAAGATGATAAACGCTATGTGGATTATGTTGGTAGCTGGGGTCCGATGATTCTGGGTCATGCACATCCGGAAGTTCTGGATGCAGTTCGTCGTCAGCTGGAACATGGTCTGAGCTATGGTGCACCGACCGCACTGGAAGTTGAAATGGCAGATCTGGTTTGTAGCCTGGTTCCGAGCATGGATATGGTTCGTATGGTTAGCAGCGGCACCGAAGCAACCATGAGCGCAATTCGTCTGGCACGTGGTTATACCGGTCGTGATGACATTATCAAATTCGAAGGTTGCTATCATGGTCATAGCGATAGCCTGCTGGTTAAAGCAGGTAGCGGTGCACTGACCTTTGGTGTTCCGAATTCACCGGGTGTTCCGGCAGATTTTGCCAAACATACCCTGACCCTGCCGTTTAATGATCTGGAAGCAGTTGATAAAGCACTGGATGAATGTGGTCAGAATGTTGCCTGTATTATTGTTGAACCGGTTGCCGGTAATATGAATTGTGTTCCGCCTGCACCGGGTTTTCTGCAGGGTCTGCGTAAAGCATGTGATAAACATGGTGTTGTGCTGATCTTTGATGAAGTGATGACCGGTTTTCGTGTTCATCTGGGTAGCGCACAGGCACTGTATGGTGTTACACCGGATCTGAGCACCTTTGGTAAAATCATTGGTGGTGGTATGCCGGTTGGTGCCTTTGGTGGTAAACGTGAAATTATGCAGCAGATTAGTCCGCTGGGTCCGGTTTATCAGGCAGGCACCCTGAGCGGTAATCCGCTGGCAATGGCAGCAGGTCTGACCACCCTGAAACTGATTAGCCGTCCTGGTTTTCATGATGAACTGACCGATTATACCAGCCGTATGCTGCAGGGCCTGCAAGAACGTGCAGATGCAGCAGGTATTCCGTTTGTTACCACCCAGGCAGGCGCAATGTTTGGTCTGTATTTTTCAGGTGCAGATGATATTGTTACCTTCGCAGATGTTATGGCAAGTGATGCAGAACGTTTTAAACGCTTTTTTCACCTGATGCTGGATGGTGGTGTTTATCTGGCACCGAGCGCCTATGAAGCAGGTTTTACCAGCATTGCACATGGTGATACCGAACTGAAACTGACCCTGGATGCAGCAGAACGTGCCTTTGCAGCACTGAAATAAGGATCC
